# Lymphotoxin **β** receptor and tertiary lymphoid organs shape acute and chronic allograft rejection

**DOI:** 10.1172/jci.insight.177555

**Published:** 2024-07-02

**Authors:** Gang Zhang, Neda Feizi, Daqiang Zhao, Latha Halesha, Amanda L. Williams, Parmjeet S. Randhawa, Khodor I. Abou-Daya, Martin H. Oberbarnscheidt

**Affiliations:** 1 Department of Surgery, Thomas E. Starzl Transplantation Institute, Pittsburgh, Pennsylvania, USA.; 2 Center of Organ Transplantation, Xiangya Hospital, Central South University, Changsha, Hunan, China.; 3 Division of Transplant Pathology, Department of Pathology, University of Pittsburgh School of Medicine, Pittsburgh, Pennsylvania, USA.; 4 Department of Immunology, University of Pittsburgh, Pittsburgh, Pennsylvania, USA.

**Keywords:** Immunology, Transplantation, Cellular immune response, Organ transplantation, T cells

## Abstract

Solid organ transplantation remains the life-saving treatment for end-stage organ failure, but chronic rejection remains a major obstacle to long-term allograft outcomes and has not improved substantially. Tertiary lymphoid organs (TLOs) are ectopic lymphoid structures that form under conditions of chronic inflammation, and evidence from human transplantation suggests that TLOs regularly form in allografts undergoing chronic rejection. In this study, we utilized a mouse renal transplantation model and manipulation of the lymphotoxin αβ/lymphotoxin β receptor (LTαβ/LTβR) pathway, which is essential for TLO formation, to define the role of TLOs in transplantation. We showed that intragraft TLOs are sufficient to activate the alloimmune response and mediate graft rejection in a model where the only lymphoid organs are TLOs in the allograft. When transplanted to recipients with a normal set of secondary lymphoid organs, the presence of graft TLOs or LTα overexpression accelerated rejection. If the LTβR pathway was disrupted in the donor graft, TLO formation was abrogated, and graft survival was prolonged. Intravital microscopy of renal TLOs demonstrated that local T and B cell activation in TLOs is similar to that observed in secondary lymphoid organs. In summary, we demonstrated that immune activation in TLOs contributes to local immune responses, leading to earlier allograft failure. TLOs and the LTαβ/LTβR pathway are therefore prime targets to limit local immune responses and prevent allograft rejection. These findings are applicable to other diseases, such as autoimmune diseases or tumors, where either limiting or boosting local immune responses is beneficial and improves disease outcomes.

## Abstract 2

The presence of intragraft lymphotoxin β receptor signaling and tertiary lymphoid organs promotes allograft rejection via activation of local immune responses.

## Introduction

In solid organ transplantation, immunosuppressive therapy has substantially improved short-term organ allograft survival by reducing acute rejection rates. However, chronic rejection — mediated by T cells, antibodies, or both — has not markedly declined in incidence and remains an important obstacle to long-term allograft survival (1, 2). Further understanding of the pathophysiology of chronic rejection is therefore necessary.

A likely important contributor to the pathogenesis of chronic rejection is the formation of tertiary lymphoid organs (TLOs) within the graft. TLOs are ectopic lymphoid structures resembling lymph nodes that arise in chronically inflamed tissues by a process called lymphoid neogenesis (3). Pathognomonic features of TLOs include distinct T cell zones, B cell zones, and high endothelial venules (HEVs), normally not found outside lymph nodes and Peyer’s patches. In the nontransplant setting, TLOs have been described in autoimmunity, chronic infection, atherosclerosis, and cancer (4). They correlate with disease severity, except in cancer, where they portend better prognosis (5, 6). In transplantation, they have been extensively documented in heart, kidney, and lung allografts in both laboratory animals and humans and are associated with chronic rejection and shorter allograft survival (7–10). For example, 78% of mouse heart allografts undergoing chronic rejection and up to 95% of human renal allograft explants due to chronic rejection have features of lymphoid neogenesis (7, 8). Some reports have also demonstrated roles in tolerance maintenance in mouse models of lung transplantation, where Tregs seem to exert their regulatory function in TLOs in recipients treated with costimulatory blockade (11). Recent work by Rosales et al. has described the presence of Treg-rich organized lymphoid structures (TOLSs) in a kidney transplantation model in mice using a specific donor — recipient stain combination (12). These structures, contrary to TLOs, do not contain HEVs, reflected by lack of peripheral node addressin (PNAd) expression. TOLSs have been shown to be important for long-term renal allograft survival, which is dependent on Tregs and can develop in the absence of secondary lymphoid tissue. Although these studies outline specific functions of TLOs in different disease models, and associations with specific disease outcomes, cause-effect experiments delineating the contribution of TLOs to allograft rejection are sparse.

The lymphotoxin αβ/lymphotoxin β receptor (LTαβ/LTβR) pathway is important for lymphoid neogenesis. The ligands for LTβR are the heterotrimer LTα1β2 and LIGHT, while the homotrimer LTα3 can bind to other members of the TNF receptor superfamily (TNFR1, TNFR2, and HVEM). Signaling through LTβR activates the NF-κB pathway as well as the JNK pathway. The alternative pathway of NF-κB activation involves activation p100, which is dependent on IKKα and NIK and is the major LTβR pathway responsible for lymph node development. This is evident by the absence of secondary lymphoid tissue in LTβR-, IKKα-, and NIK-deficient mice.

Studies utilizing skin transplantation in recipients that do not have secondary lymphoid organs have demonstrated that skin containing TLOs can mediate allograft rejection at the same site or of skin transplanted elsewhere (13).

Despite these different roles of TLOs in immunity and allograft rejection, several questions remain: Are TLOs contributing to allograft rejection? What immune functions do TLOs support in vivo?

In this article, we utilize a renal allograft transplantation model in mice and manipulation of the LTβR/LTαβ pathway to elucidate the role of TLOs in allograft rejection. Moreover, we developed an intravital microscopy model to visualize immune cell interactions in renal TLOs to investigate if TLOs support activation of T and B cells. We found that TLOs are sufficient for renal allograft rejection, that they contribute to rejection even in the presence of lymph nodes, and that disrupting the LTβR pathway prolongs allograft survival. Intravital microscopy showed that TLOs support T and B cell activation.

## Results

### TLOs are sufficient for renal allograft rejection.

**Figure 1:**
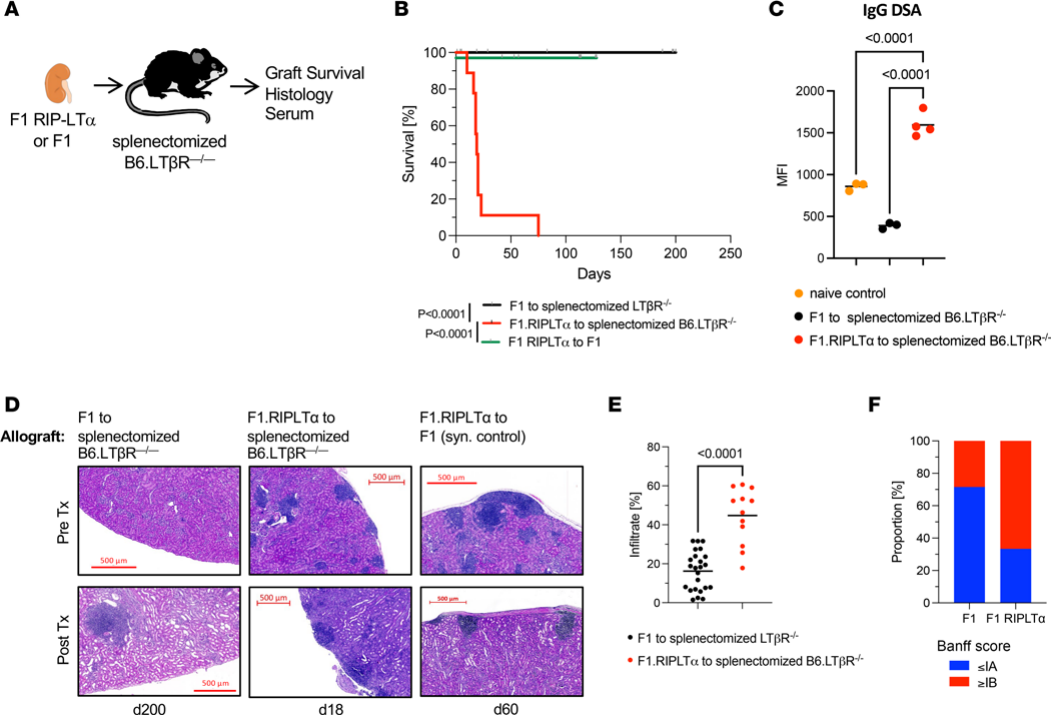
TLOs are sufficient for renal allograft rejection. (**A**) F1 (*n* = 7) or F1.RIP-LTα (*n* = 6) donor kidneys were transplanted to splenectomized B6 LTβR-KO mice and graft survival was monitored. (**B**) Kaplan-Meier curve of graft survival. Median survival time (MST) of F1.RIP-LTα grafts was 23 days. Recipients of F1 allografts were sacrificed on day 200 with functioning graft. F1 recipients of syngeneic F1-RIP-LTα grafts were sacrificed on day 90 with functioning grafts. Sample size, *n* = 6–7. *P* < 0.0001, determined by log-rank (Mantel-Cox) test. (**C**) Flow cytometric assay assessing serum IgG DSA of graft recipients. *P* values were determined by 1-way ANOVA with multiple comparisons. (**D**) Representative images of H&E-stained sections of allograft tissue at indicated time points after transplantation. Pretransplant native kidney images from the same donor strain shown for reference. Scale bars: 500 μm. (**E**) Histological quantitation of immune infiltration. *P* values were determined by 1-way ANOVA with multiple comparisons. (**F**) Banff rejection scores of histology procured at time of graft failure. *P* values were determined by Fisher’s exact test.

To investigate whether renal TLOs are sufficient to initiate an alloimmune response and cause graft rejection, we used splenectomized LTβR-deficient (LTβR-KO) mice as recipients of F1 (B6 × BALB/c, CB6F1) or rat insulin promoter-lymphotoxin alpha transgenic CB6F1 (F1-RIP-LTα) kidneys (Figure 1A). LTβR-KO mice do not have lymph nodes or Peyer’s patches, and after splenectomy they are devoid of all secondary lymphoid organs. Secondary lymphoid organs are necessary to mount an alloimmune response and reject an allograft (14). F1-RIP-LTα mice express lymphotoxin α (LTα) under control of the rat insulin promoter and develop spontaneous TLOs in the pancreas, skin, and kidney at 4–6 months of age (15). F1-RIP-LTα donor kidneys therefore contain preformed TLOs at the time of transplantation, while F1 WT kidneys do not. In this model, the only lymphoid tissue present is the TLO in the donor graft. To rule out that the presence of inflammatory TLO or LTα overexpression in the donor graft has a functional consequence independent of an alloimmune response, we performed syngeneic F1-RIP-LTα kidney transplants to F1 recipients as controls. As shown in Figure 1B, F1 allografts survived beyond 200 days, while F1-RIP-LTα grafts containing TLOs were rejected, with a mean survival time (MST) of 23 days. Syngeneic F1-RIP-LTα grafts were maintained beyond day 90. Donor-specific antibody (DSA) measurements in the serum on day 50 showed a lack of DSAs in recipients of WT allografts, while IgG DSA was present in recipients that received F1-RIP-LTα allografts, suggesting that TLOs provide a place for B cell activation and antibody formation (Figure 1C). Histopathology (Figure 1D) demonstrates the presence of TLOs before transplantation as well as at time of rejection in F1-RIP-LTα allografts. F1 allografts showed less infiltration and lower proportions of severe rejection (Banff scores > 1B) (Figure 1, E and F) on day 200 but are characterized by the presence of lymphoid aggregates around small arteries. Syngeneic F1-RIP-LTα grafts displayed presence of TLOs before and after transplantation, but no other immune infiltrate (Figure 1D), and surpassed rejection time of the F1-RIP-LTα allografts transplanted to LTβR^–/–^ recipients. TOLSs have been previously documented, notably even in the absence of secondary lymphoid tissue (12). To further characterize the lymphoid aggregates present in allografts, we performed IF staining for T, B, FoxP3, and PNAd in both F1 and F1-RIP-LTα grafts. As shown in Supplemental Figure 1 (supplemental material available online with this article; https://doi.org/10.1172/jci.insight.177555DS1), lymphoid aggregates in F1 allografts are characterized by T and B cell areas, the presence of FoxP3^+^ Tregs, and the absence of PNAd, fulfilling the main criteria for TOLS. Lymphoid aggregates in F1-RIP-LTα grafts also contained T and B cell areas but lacked FoxP3^+^ Tregs. In addition, PNAd staining was present in these structures, a hallmark of inflammatory TLOs. The long-term life-sustaining function of the syngeneic F1-RIP-LTα kidney grafts suggests that graft failure in allogeneic kidney transplants is a consequence of rejection rather than the mere presence of TLOs or LTα overexpression in the graft. These data demonstrate that preformed TLOs are sufficient for allograft rejection and support a full alloimmune response with T and B cell activation/DSA production.

### TLOs accelerate renal allograft rejection.

**Figure 2:**
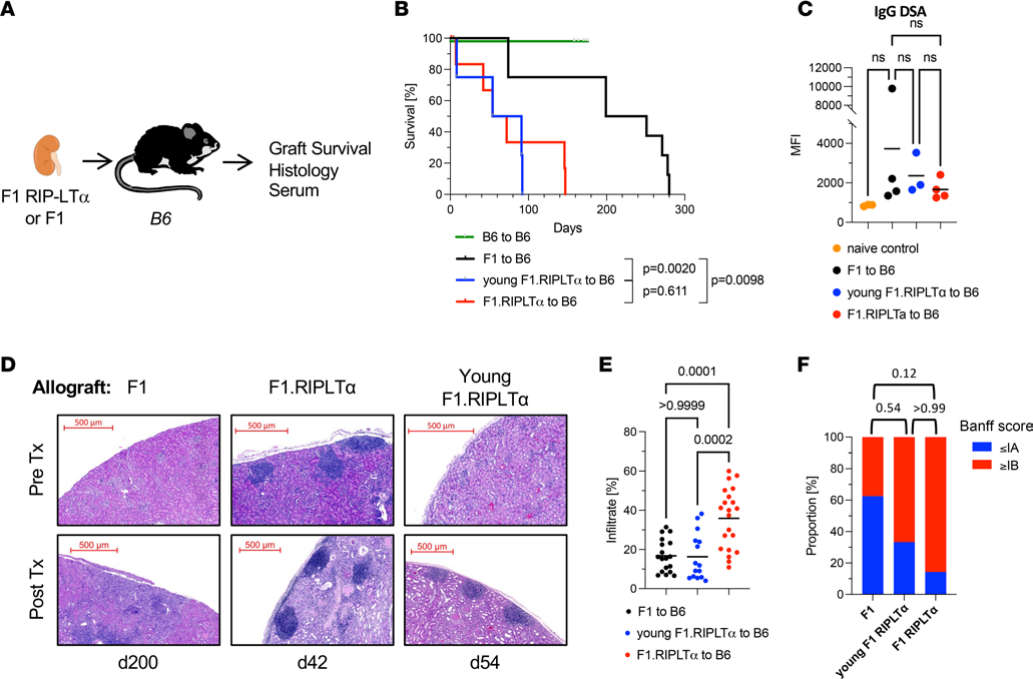
Preformed TLOs accelerate renal allograft rejection. (**A**) F1 or F1.RIPLTα donor kidneys were transplanted to B6 recipients. Young F1.RIPLTα donors were 8 weeks old, and kidneys did not contain TLOs at the time of transplantation. (**B**) Kaplan-Meier curve of graft survival. F1 MST = 225 days (*n* = 8), F1.RIPLTα MST = 63 days (*n* = 6), young F1.RIPLTα MST = 72.5 days (*n* = 4). Syngeneic B6 grafts shown as controls (MST >200 days, *n* = 9). *P* values were determined by log-rank (Mantel-Cox) test. (**C**) Flow cytometric assay assessing serum IgG DSA of graft recipients 60 days after transplantation. *P* values were determined by 1-way ANOVA with multiple comparisons. (**D**) Representative images of H&E-stained sections of allograft tissue at indicated time points after transplantation. Pretransplant native kidney images from the same donor strain shown for reference. Scale bars: 500 μm. (**E**) Histological quantitation of immune infiltration. *P* values were determined by 1-way ANOVA with multiple comparisons. (**F**) Banff rejection scores of histology procured at the time of graft failure. *P* values were determined by Fisher’s exact test.

We next investigated if TLOs contribute to renal allograft rejection in the presence of a normal set of secondary lymphoid tissues. We transplanted either F1 or F1-RIP-LTα kidneys to WT B6 recipients and monitored allograft survival (Figure 2A). As LTα can bind as a heterotrimer, LTα1β2, to LTβR and as a homotrimer, LTα3, to TNFR family members, mediating inflammatory signals, we also transplanted F1-RIP-LTα kidneys from young donors (8 weeks old), in which no TLOs had formed at the time of transplantation, but LTα was overexpressed. Allograft survival of F1-RIP-LTα kidneys was significantly shorter (MST = 63 days) than survival of F1 allografts (MST = 225 days), indicating that preformed TLOs in the graft accelerate allograft rejection (Figure 2B). Renal allografts from young F1-RIP-LTα donors were also rejected significantly earlier (MST = 72.5 days) than F1 allografts. No significant differences were detected in DSA formation (Figure 2C). Histopathology at the time of rejection demonstrated the presence of TLOs in all allografts. F1-RIP-LTα grafts displayed prominent TLOs before and after transplantation. In young F1-RIP-LTα donor grafts, only occasional lymphoid aggregates were present before transplantation, but TLO developed quickly after transplantation (Figure 2D), which makes it impossible to separate the inflammatory effects of LTα from TLO functions. F1 allografts demonstrated development of TLO with HEVs (PNAd expression) (Supplemental Figure 2) at the time of rejection, suggesting that de novo TLO formation in WT grafts requires a longer time to occur (Figure 2D). Histological quantitation of the cellular infiltrate (Figure 2E) and Banff rejection scores (Figure 2F) confirmed that the presence of preformed or rapidly forming TLOs in the allograft leads to a larger immune infiltrate and higher Banff scores, reflecting the differences in median survival time. These data support that TLO and LTα-LTβR signaling contribute to chronic allograft rejection in WT recipients.

### Blocking donor LTβR signaling prolongs allograft survival.

**Figure 3:**
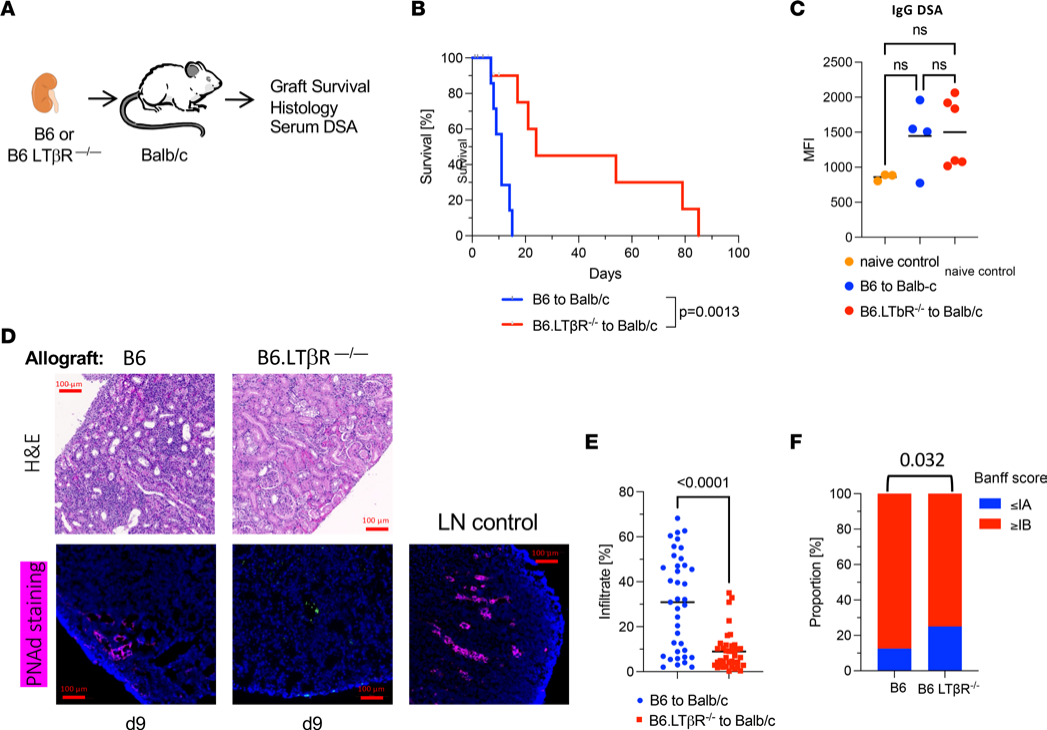
Blocking donor LTβR signaling prolongs allograft survival. (**A**) B6 or B6.LTβR-KO donor kidneys were transplanted to BALB/c recipients. (**B**) Kaplan-Meier curve of graft survival. B6 MST = 11 days (*n* = 7), B6.LTβR-KO MST = 24 days (*n* = 7). *P* values were determined by log-rank (Mantel-Cox) test. (**C**) Flow cytometric assay assessing serum IgG DSA of graft recipients 9 days after transplantation. *P* values were determined by 1-way ANOVA with multiple comparisons. (**D**) Top row: Representative images of H&E-stained sections of allograft tissue at indicated time points after transplantation. Bottom row: Representative immunofluorescence images with PNAd (magenta) and DAPI (blue) staining of allograft tissue on day 9 after transplantation. Lymph node shown as control. Scale bars: 100 μm. (**E**) Histological quantitation of immune infiltration. *P* values were determined by 1-way ANOVA with multiple comparisons. (**F**) Banff rejection scores of histology procured at time of graft failure. *P* values were determined by Fisher’s exact test.

To further elucidate the role of TLOs in allograft rejection, we performed transplantation survival experiments in which donor LTβR signaling is disrupted. LTβR is critically important for secondary lymphoid organ and TLO formation and binds two different ligands, the heterotrimeric LTα1β2 and LIGHT. While the heterotrimer LTα1β2 only binds to LTβR, the LTα3 homotrimer has inflammatory properties similar to TNF-α and binds to TNFRI, TNFRII, and HVEM but not to LTβR. LTα3 has been associated with autoimmunity and inflammatory diseases. In this model, we were therefore able to separate the proinflammatory effects of LTα3 signaling from the effects of blocking LTβR signaling. We used B6 WT or B6 LTβR-KO donor grafts transplanted to BALB/c recipients (Figure 3A). The B6-to-BALB/c kidney transplantation model results in acute rejection of renal allografts. As LTβR expression on stromal cells is essential for lymphoid neogenesis, the absence of LTβR on donor graft tissue prevented intragraft TLO formation and inflammatory signals mediated through LTβR. As shown in Figure 3B, B6 WT allografts were quickly rejected (MST = 11 days), while B6 LTβR-KO allografts survived significantly longer (MST = 24 days). No statistically significant difference in IgG DSA production in the B6 WT allograft recipients compared with B6 LTβR-KO graft recipients was observed (Figure 3C). Histopathology of a subset of grafts procured on day 9 after transplantation showed more severe immune cell infiltration in B6 WT allografts compared with B6 LTβR-KO allografts (Figure 3D). Immunofluorescence for PNAd showed presence of HEV in B6 allografts but not in B6 LTβR-KO allografts, indicating that B6 WT renal allografts promote lymphoid neogenesis very early after transplantation (Figure 3D). Quantitation of the immune infiltrate at the time of rejection revealed a significant higher infiltration in B6 WT allografts compared with B6 LTβR-KO allografts (Figure 3E), and Banff rejection scores at the time of rejection were significantly higher (>IB compared with <IA) in B6 allografts compared with B6 LTβR-KO allografts (Figure 3F).

### Renal TLOs support naive immune cell activation.

**Figure 4:**
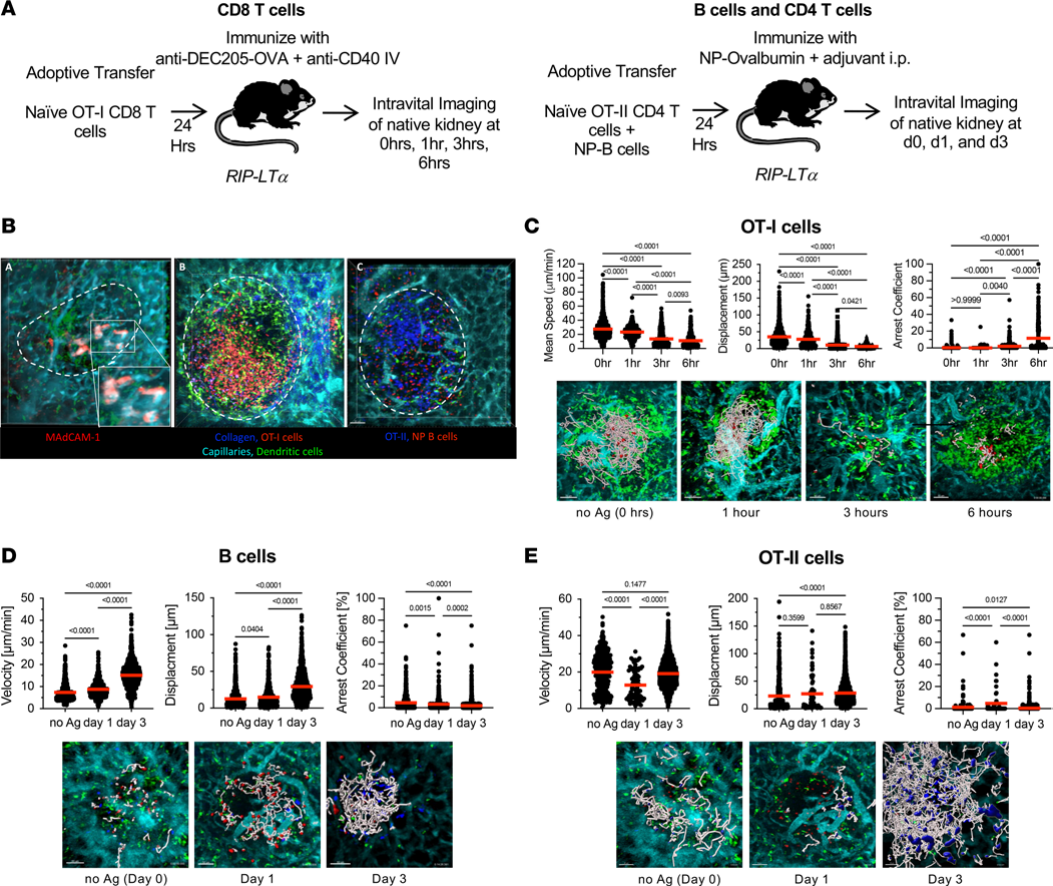
Intravital microscopy of TLO and immune cell interactions. (**A**) Experimental design of imaging experiments: 10 million OT-I dsRed CD8 T cells or 10 million OT-II CFP CD4^+^ T cells and 30 million NP-specific B1.8 B cells labeled with CellTracker Red were adoptively transferred to naive B6.RIPLTα mice 1 day before imaging. TLOs were imaged at time 0 (before antigen administration) and at indicated times after immunization. Sample size, *n* = 3 animals per time point, *n* = 3–6 time lapse recordings per animal. (**B**) Intravital microscopy images depicting Left: MAdCAM-1 staining limited to a TLO (dotted line) with surrounding normal kidney tissue. Capillaries (cyan), dendritic cells (green). Middle: TLO with dendritic cells (green) and OT-I CD8 T cells (red). Collagen fibers (blue, second harmonic signal). Right: TLO with OT-II CD4^+^ T cells (blue), B cells (red), and dendritic cells (green). Collagen (blue). Scale bar: 50 µm. (**C**) CD8 T cell motility parameters. Top: Quantitation of mean speed, displacement, and arrest coefficient of CD8 OT-I T cells at indicated time points. Bottom: Representative images depicting tracks of OT-I CD8 T cells at different time points. *P* values were determined using 1-way ANOVA with Tukey’s multiple comparison test. Scale bar: 50 µm. (**D**) Analysis of B cell motility parameters. Top: Quantitation of mean speed, displacement, and arrest coefficient of B1.8 B cells at indicated time points after antigen administration. Bottom: Representative images with total B cell track lengths depicted in white at indicated time points. Scale bar: 50 µm. (**E**) Analysis of OT-II motility parameters. Top: Quantitation of mean speed, displacement, and arrest coefficient of OT-II CD4^+^ T cells at indicated time points. Bottom: Representative images depicting total CD4^+^ T cell track lengths in white at indicated time points. *P* values were determined using 1-way ANOVA with Tukey’s multiple comparison test. Scale bar: 50 µm.

To further investigate the function of TLOs, we developed an intravital microscopy model to study cell-cell interactions in vivo in TLOs under the kidney capsule. This allowed us to compare immune cell interactions in TLOs to those observed in lymph nodes. We imaged kidneys of bone marrow chimeric CD11c-YFP B6 RIP-LTα mice, where we could identify TLOs by (a) the lack of normal kidney structure (capillaries) and (b) the accumulation of CD11c-YFP^+^ dendritic cells. To clearly define the time point of antigen introduction, we utilized transgenic OT-I r(ed fluorescent protein dsRed [dsRed]) and OT-II (cyan fluorescent protein [CFP]) T and 4-hydroxy-3-nitrophenyl acetyl (NP)-specific (CellTracker Red) B cells, imaged at time 0 and after 1, 3, 6, 24 and 72 hours after immunization with either anti-DEC-205 (OT-I T cells) or NP-ovalbumin (OT-II and B cells) (Figure 4A). As shown in Figure 4B, TLOs can be identified by MAdCAM-1 expression (HEV); accumulation of dendritic cells; presence of naive T or B cells, which are absent in normal surrounding kidney tissue; and lack of normal kidney tissue structure. Naive T and B cells accumulate in distinct zones (Figure 4B). We first evaluated motility parameters of OT-I T cells before and after immunization with anti–DEC205-OVA and FGK4.5 (16). OT-I T cells displayed a reduction in mean speed and displacement after immunization and an increased arrest coefficient over time (Figure 4C and Supplemental Video 1), which is reflected in total track length shown in the bottom of Figure 4C. To investigate B cell activation, we transferred NP-specific B cells (labeled with CellTracker Red CMTPX [Invitrogen, catalog C34552]) and OT-II T cells (CFP) and performed imaging at day 0 and days 1 and day 3 after immunization with NP-ovalbumin and adjuvant. For B cells, we observed an increase in motility and displacement after immunization, which is similar to observations made in lymph nodes and consistent with B cell activation (Figure 4D and Supplemental Video 2) (17, 18). CD4 OT-II T cells were imaged at the same time points and displayed lower mean speed and increased arrest coefficient on day 1 after immunization with increased motility parameters on day 3 (Figure 4E and Supplemental Video 3) and associated changes in total track length (Figure 4E, bottom). The observations in T cells are indicative of stable cell-cell interactions needed for activation and similar to motility changes that have been observed during T cell activation in lymph nodes (19, 20).

## Discussion

The formation of TLOs through the process of lymphoid neogenesis has long been associated with chronic inflammatory conditions where antigen persists (21). This is the case during chronic infections, autoimmune diseases, and organ transplantation (22–25). The discovery that many cancers also promote TLO formation and that the presence of TLOs often is a predictor of better outcomes has led to a resurgence of interest in TLOs (26). TLOs have immunomodulatory effects, they can either promote immunity or can be associated with immune regulation (11, 27, 28). In transplantation, TLOs have been associated with both, chronic rejection outcomes and graft acceptance, at least in animal models (7, 11, 13, 27). In this study, we have performed cause-effect experiments to further define the role of TLOs in acute and chronic rejection in a mouse model of kidney transplantation. We not only defined the role of TLOs by manipulating the LTαβ/LTβR pathway in survival experiments, but also developed a model of intravital microscopy to capture for what we believe to be the first time the cellular events and interactions in TLOs, similar to what has been studied in lymph nodes.

We demonstrate that TLOs are sufficient to mediate allograft rejection in recipients that do not have secondary lymphoid tissue and do not reject allografts in the absence of TLOs. This highlights that TLOs are fully functional lymphoid organs that are capable of providing the environment needed for activation of an adaptive immune response in a model of vascularized, solid organ transplantation. This includes the activation of B cells and production of DSAs, which do not develop if WT, non-TLO containing, allografts are transplanted. The chronic kidney transplantation model used in our studies is not dependent on DSAs, of which we were only able to detect low levels in the serum, independent of the presence of preformed graft TLOs. The significance of B cell activation and DSA production in graft TLOs needs to be further investigated.

We observed that F1 allografts, although maintained long-term and not undergoing rejection, contained lymphoid aggregates that resemble the TOLSs that have been previously reported (12). Our data confirm that TOLSs can form in a donor-recipient strain combination where the recipient lacks secondary lymphoid tissue, as first described by Rosales et al. (12). These aggregates were characterized by the presence of Tregs, the absence of HEVs expressing PNAd, and a location around a central blood vessel. These aggregates were not present when F1 allografts were transplanted to B6 WT recipients, where we observed formation of inflammatory TLOs over time. The formation of TOLSs in renal allografts transplanted to LTβR^–/–^ recipients offers an opportunity for further research to elucidate what conditions and mechanisms govern their formation and function.

This study demonstrates that TLOs accelerate allograft rejection in the presence of a normal set of secondary lymphoid tissues. While WT F1 allografts also demonstrated TLOs at the time of rejection, there was a marked difference in rejection tempo if TLOs were present at the time of transplantation, suggesting a local contribution of TLOs in the alloimmune response. A caveat of TLO studies is that the function of TLOs is linked to local inflammation, as a chronic inflammatory environment is essential to provide the conditions necessary for the development of TLOs. This is also applicable to the model utilized in this study. The RIP-LTα model causes local overexpression of the inflammatory mediator LTα, which then provides the signal for TLO formation. We attempted to address this by transplanting donor kidneys from young F1 RIP-LTα mice that did not harbor TLOs at the time of transplantation, but the histological presence of TLOs after accelerated allograft rejection confirmed that the function of TLOs and inflammatory signals could not be separated in this experiment. A separate TLO-independent function of LTα can therefore not be ruled out.

In an acute kidney rejection model (B6 to BALB/c), disrupting the LTβR pathway in donor allografts led to prolonged allograft survival compared with that of WT B6 donors. Prolongation of allograft survival took place with intact LTα3 and TNF-α signaling pathways that mediate inflammatory signals, which highlights the importance of the LTβR pathway in allograft rejection. The outcome of prolonged allograft survival cannot be attributed solely to lymphoid neogenesis, as LTβR signaling involves activation of both the NF-κB and JNK pathways, which play roles not only in lymphoid neogenesis, but also mediate inflammatory signals. It is therefore possible that not only the prevention of TLO formation is causative for better allograft survival, but that the absence of inflammatory signals mediated though LTβR also contributes to this outcome. However, physiologically, the presence of TLOs is intrinsically linked to inflammatory signals, making TLOs and the LTβR pathway relevant targets to improve allograft outcomes.

The intravital microscopy TLO data in this model suggest that productive cell-cell interactions, leading to activation of naive T cells, are taking place in TLOs. Together with the survival data presented, TLOs are therefore likely to contribute to local immune activation and maintenance in this model. As the cell-cell interactions and motility parameters observed in TLOs are similar to what has been described in lymph nodes, it is likely that TLOs support similar immunological functions, including promoting regulatory functions under the appropriate conditions. It is promising that interfering with the LTβR pathway in the donor organ can delay allograft rejection significantly in an acute kidney graft rejection model in mice. This work used transplantation as a model to investigate the function of TLOs, but the results are equally relevant to autoimmunity, cancer, and other chronic inflammatory conditions.

## Methods

### Sex as a biological variable.

Both sexes of mice were used, but males were preferred for the transplantation procedure due to size and anatomy. Previous studies have not identified sex differences in allograft rejection beyond the known H-Y minor histocompatibility Ag in the absence of an MHC mismatch. The findings obtained in this study are expected to be relevant to both sexes.

### Study design.

Three biological replicates (3 individual transplant recipients) per group were included in each experiment. Experiments were repeated once, resulting in a total of up to 6 biological replicates. Sample sizes were based on prior observations that 3–6 biological replicates were sufficient to discern statistically significant differences between groups, with observed effect sizes >0.5. Prospective exclusion criteria were transplant recipient death within the first 7 days after transplantation (technical failure) and urinary obstruction (censored data points). All other data points were included, and no outliers were excluded. All end points were prospectively selected. It was not possible to blind the study because of the need to identify donors and recipients. Histopathological scoring was performed by masked investigators.

### Animals.

B6.CD45.2 (C57BL/6J; Thy1.2, CD45.2), B6.CD45.1 (B6.SJL-*Ptprc^a^Pepc^b^*/BoyJ, Thy1.2, CD45.1), DsRed [B6.Cg-Tg(CAG-DsRed*MST)1Nagy/J], BALB/c CD45.1 (CByJ.SJL(B6)-Ptprca/J), F1 (CB6F1/J), and B6 CD11c-YFP (B6.Cg-Tg(Itgax-Venus)1Mnz/J) mice were from The Jackson Laboratory. B6.CD45.1 (B6.SJL-*Ptprc^a^Pepc^b^*/BoyCrl) mice were from Charles River Laboratories. B6 RIP-LTα mice were maintained and bred at the University of Pittsburgh and were originally from Nancy Ruddle (Yale University, New Haven, Connecticut, USA). B6 B18 NP-specific B cell–transgenic mice were from Mark Shlomchik (Department of Immunology, University of Pittsburgh). B6 OT-II (B6.Cg-Tg(TcraTcrb)425Cbn/J) mice (The Jackson Laboratory) were crossed to B6.CFP (B6.129(ICR)-Tg(CAG-ECFP)CK6Nagy/J) mice and maintained on a B6 Rag-deficient background. B6 OT-I mice (C57BL/6-Tg[TcraTcrb]1100Mjb/J; CD45.2) were obtained from The Jackson Laboratory and maintained on a RAG^–/–^ DsRed background. B6 LTβR^–/–^ were maintained and bread at the University of Pittsburgh and were originally received from the University of Chicago, Chicago, Illinois, USA. Transplant recipients were 8–12 weeks old, and RIP-LTα mice were used at age 5–8 months when TLOs were consistently present. Some experiments utilized young RIP-LTα mice (8 weeks).

### Kidney transplantation and nephrectomy.

Mouse kidney transplants were performed as previously described (29). Recipient native kidneys were removed during the transplantation procedure. Allograft rejection was monitored by visual observation of recipients for signs of uremia (lethargy, decreased mobility, and ruffled hair) or death.

### Bone marrow chimeras.

CD11c-YFP bone marrow chimeras were generated by irradiating B6.RIP-LTα mice with 10 Gy followed by adoptive transfer 10 × 10^6^ BM cells i.v. from CD11c-YFP. Mice received sulfatrim food for 14 days after irradiation. Reconstitution was confirmed 56 days after bone marrow transplantation by visualizing tissue turnover of tissue dendritic cells using YFP fluorescence.

### Histological analysis and Immunofluorescence staining.

Kidney allograft tissue was fixed in formalin, paraffin-embedded, sectioned, and stained with H&E, Masson’s trichrome, and periodic acid–Schiff stain (Magee-Women’s Research Institute Histology and Microimaging Core, University of Pittsburgh). For Immunofluorescence, cryosections were stained with primary antibodies for 16 hours at 4°C. Following avidin/biotin blocking, slides were incubated with biotinylated secondary antibody for 30 minutes at room temperature and then streptavidin-conjugated quantum dots for 30 minutes at room temperature. For FFPE tissue, sections were deparaffinized, and antigen retrieval was performed at pH 6 for 30 minutes at 96°C (Target Retrieval Solution pH6, Agilent), followed by blocking with FBS and 5% rat serum. Sections were then stained with primary antibodies, including PNAd (biotin, clone MECA-79, BioLegend), CD3 (rabbit, catalog A0452, Agilent), and B220 (Alexa Fluor 488, clone RA3-6B2, eBioscience), and secondary antibodies, including streptavidin-Alexa Fluor 647 and goat anti-rabbit IgG H+L (Invitrogen, catalog A-11012, Alexa Fluor 594). FoxP3 staining was performed after additional 1% Triton X-100 incubation for 30 minutes, followed by FoxP3-Alexa Fluor 647 (BioLegend, clone 150D). DAPI was used to visualize nuclei. Stained sections were mounted in EcoMount (Biocare Medical). All slides were scanned on a Zeiss Axioscan.Z1 with a ×20 objective and analyzed in QuPath (30). A pixel classifier was trained in QuPath to quantitate immune infiltration per kidney section at time of rejection, using H&E-stained sections. Histological sections of allografts were scored according to Banff classification.

### Donor-reactive antibody detection.

DSAs were detected by incubating recipient serum with donor splenocytes and detecting bound antibodies with anti-mouse IgG-FITC antibody (Life Technologies, catalog 11-4011-85). Briefly, donor F1 splenocytes were incubated with 20% FBS for 20 minutes at room temperature to block nonspecific binding. Recipient serum (25 μL) was added to 0.5 × 10^6^ donor splenocytes and incubated on ice for 1 hour. Cells were washed, and surface staining for CD3-PE (eBioscience, clone 145-2C11), B220-eF450 (eBioscience, clone RA3-6B2), and anti-IgG was performed. Samples were acquired on a BD Fortessa or Cytek Aurora spectral cytometer. Alloantibody binding was assessed on T cells and MFI reported (Supplemental Figure 3).

### Two-photon intravital imaging.

Multiphoton intravital microscopy was performed on transplanted kidneys. Multiphoton intravital microscopy was performed on native kidneys. OT-I and OT-II T cells and NP-B cells were isolated from spleens using a magnetic bead isolation kit (EasySep Mouse T Cell Isolation Kit, Stem Cell Technologies, catalog 19851, or EasySep Mouse Pan-B Cell Isolation Kit, Stem Cell Technologies catalog 19844). B cells were fluorescently labeled with 2.5 µM CellTracker Red (Invitrogen, catalog C34552). 10 million cells of each indicated cell population were adoptively transferred i.v. to F1-RIP-LTα CD11c-YFP bone marrow chimeric mice 24 hours prior to imaging. Antigen was administered by injecting 250 µg anti-DEC205-OVA fusion antibody i.v. (gift from Warren Shlomchik, University of Pittsburgh, Pittsburgh, Pennsylvania, USA) or i.p. injection of 50 µg NP-ovalbumin (Santa Cruz, catalog sc-396355) in Alhydrogel adjuvant 2% (Invivogen, catalog vac-alu-50). A custom Leica TCS SP8 Triple Beam 6 Ch NDD system containing 6 HyD detectors and two Spectra Physics femto-second pulsed lasers (MaiTai DeepSee and Insight X3) with 3 laser lines was used. The laser was tuned and mode-locked to 920 nm. The following filter sets (all from Chroma) were used: 583/22 nm (dsRed), 537/26 nm (EYFP), 483/32 nm (CFP), and 655/15 nm (Evans Blue, Sigma, catalog E2129). Microscope data were acquired with Leica LAS X v2.53. Mice were anesthetized with isoflurane and oxygen, and core body temperature maintained at 37°C with a homeothermic controller (TC-1000, CWE). Animals were kept hydrated by injecting 1 mL 5% dextrose lactated ringer’s solution s.c. every 60 minutes. Blood vessels were visualized by injecting Evans Blue (3–6 μL of 5 mg/mL stock solution [15–30 μg] diluted in PBS i.v.), and HEVs were visualized by injecting 3 μg of PE-conjugated MAdCAM-1 antibody i.v. (Biolegend, clone MECA367). The kidney was extraverted from the abdominal cavity with intact vascular connection and immobilized in a custom cup mount. A coverslip was placed on top of the kidney, and *Z*-stacks were visualized with a 25× water immersion objective (NA: 1.05) up to 70 μm below the kidney capsule. All stacks were acquired with a step size of 1 μm. Brightness and laser power were adjusted based on the imaging depth and kept below phototoxic levels. Line averaging was set to 4× at a resolution of 512 × 512 pixels using the resonance scanner. Time-lapse imaging was performed for approximately 30 minutes per location. Up to 5 different locations per kidney graft were imaged. All acquired videos were analyzed using Imaris software V9 (Oxford Instruments). Drift was corrected using dendritic cells or vasculature as a reference point. Background subtraction was performed on all channels equally.

### Statistics.

Statistical analysis was performed using Prism v.9 (GraphPad). Parametric and nonparametric tests were used as indicated in figure legends and included log-rank (Mantel-Cox) test and 1-way ANOVA with multiple comparisons. Categorical data were analyzed using Fisher’s exact test where indicated. All *P* values, regardless of statistical significance, were reported, and P values of less than 0.05 were considered significant.

### Study approval.

All animal experiments were performed with approval and under supervision of the Institutional Animal Care and Use Committee of the University of Pittsburgh (protocol 20107883; Animal Welfare Assurance, D16-00118; A3187-01).

### Data availability.

All data point values are available in the Supporting Data Values file.

## Author contributions

MHO conceived studies and designed experiments. GZ, NF, DZ, and KIA conducted experiments. GZ and DZ performed mouse kidney transplant procedures. MHO, NF, and KIA analyzed and interpreted data. LH performed immunofluorescence staining. LH and ALW performed flow cytometry and genetic typing of mice. PSR conducted the histological analysis and scoring. MHO wrote the manuscript. All authors contributed to reviewing and editing the manuscript.

## Supplementary Material

Supplemental data

Supplemental video 1

Supplemental video 2

Supplemental video 3

Supporting data values

## Version 1 07/02/2024

In-Press Preview

## Version 2 08/08/2024

Electronic publication
