# Supplementary material for: Lymphotoxin β receptor and tertiary lymphoid organs shape acute and chronic allograft rejection
Source: JCI Insight. 2024 Jul 2;9(15):e177555. doi: 10.1172/jci.insight.177555 (PMC11383591; doi:10.1172/jci.insight.177555)
Supplement: Supplemental data [file jciinsight-9-177555-s176.pdf]

**Supplemental Material**

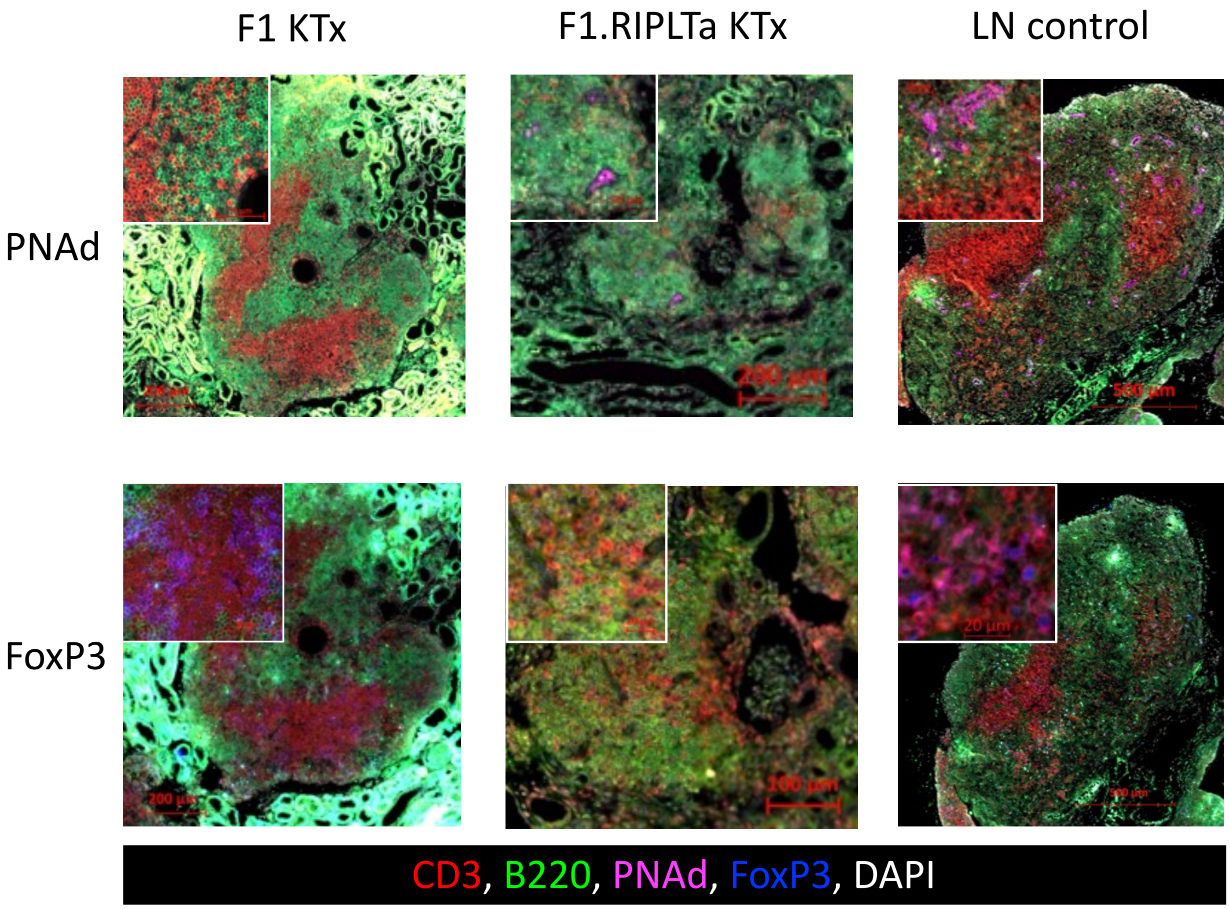


**Supplemental Figure 1**: Characterization of lymphoid aggregates in renal allografts. F1 and F1.RIPLTα KTx transplanted to B6 LTβR$^{-/-}$ recipients were stained for T cells (CD3, red), B cells (B220, green), PNAd (magenta), FoxP3 (blue), and DAPI (white). Photomicrographs show low and higher magnification of the indicated tissue. LN sections from naïve mice are shown as controls. Lymphoid aggregates in F1 KTx display T and B cells around a central blood vessel and the presence of FoxP3$^{+}$ T cells (Treg), but no PNAd staining, resembling TOLS. F1.RIPLTα KTx display T and B cell zones with PNAd expression and no FoxP3$^{+}$ T cells, resembling inflammatory TLO.

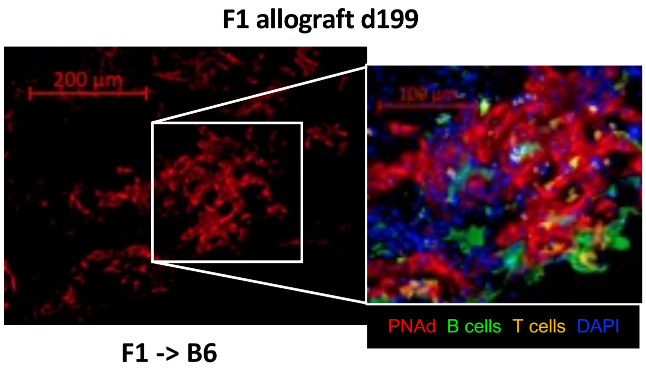


**Supplemental Figure 2:** TLO in renal allografts express PNAd. Immunofluorescence staining of an F1 allograft 199 days after transplantation. PNAd (red), B cells (CD20, red), T cells (CD3, yellow) and DAPI (blue). Photomicrograph shows PNAd staining and inset displays B, T cells and DAPI staining.

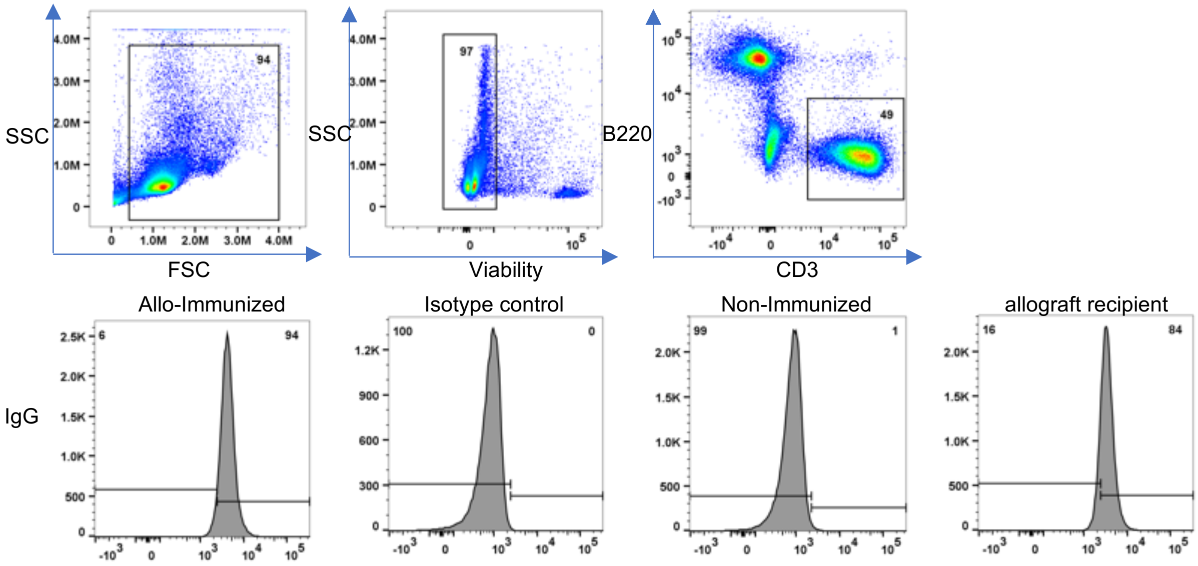


**Supplemental Figure 3:** Flow cytometry assay to detect serum donor-specific antibodies. Recipient serum was incubated with donor splenocytes and bound DSA detected with fluorochrome-conjugated anti-mouse IgG antibody. Flow cytometry gating strategy and representative histograms of anti-IgG staining shown.

**Supplemental Movie 1:** OT-I CD8 T cell activation in TLO. Composite movie of intravital imaging of TLO at indicated time points after antigen administration. Individual movies are looped for the duration of the composite movie and correspond to Figure 4C.

**Supplemental Movie 2:** B cell activation in TLO. Composite movie of intravital imaging of TLO at indicated time points after antigen administration. Individual movies are looped for the duration of the composite movie and correspond to Figure 4D.

**Supplemental Movie 3:** OT-II T cell activation in TLO. Composite movie of intravital imaging of TLO at indicated time points after antigen administration. Individual movies are looped for the duration of the composite movie and correspond to Figure 4E.
